# An examination of the psychological mechanisms between body appreciation and life satisfaction among older adults from the perspective of self-acceptance and self-perceptions of aging

**DOI:** 10.3389/fpsyg.2026.1799777

**Published:** 2026-03-19

**Authors:** Yan Lyu, Lixun Shi

**Affiliations:** 1 College of Foreign Languages, University of Shanghai for Science and Technology, Shanghai, China; 2 School of Foreign Studies, China University of Political Science and Law, Beijing, China

**Keywords:** body appreciation, life satisfaction, older adults, self-acceptance, self-perceptions of aging

## Abstract

**Background:**

Older adults are more likely to undergo changes in their physical appearance and health condition than younger people. These changes may relate to how they view their bodies and themselves, and may also be associated with differences in levels of life satisfaction. While many previous studies have focused on disease burden and functional limitations, this study examines the statistical association between body appreciation and life satisfaction from the perspective of positive body experience. It also investigates the mediating roles of self-acceptance and self-perceptions of aging in this relationship.

**Methods:**

This study surveyed 605 older adults aged 60–80 using standardized scales to measure body appreciation, self-acceptance, self-perceptions of aging, and life satisfaction. A parallel mediation model was constructed and tested.

**Results:**

After controlling for several demographic factors, body appreciation was positively related to life satisfaction. Self-acceptance played a mediating role between the two. Self-perceptions of aging did not show an independent mediating effect in the model.

**Conclusion:**

Positive body experiences and self-attitudes among older adults are positively related to higher life satisfaction. These findings provide references for the design of supportive services for older adults in community and health service settings.

## 1 Introduction

Examining the psychological underpinnings of older adults’ life satisfaction has gained importance in light of the rapidly aging population. Studies have found that life satisfaction is closely related to mental health, social participation, and health behaviors (Alumona et al., 2024; Bramhankar et al., 2023; Gu et al., 2025). So, it is necessary to further clarify related factors and possible association patterns. Prior studies suggest that body appreciation is linked to positive psychological outcomes, providing support for the role of positive body attitudes in mental health (Linardon et al., 2023). Older adults experience changes in physical function, appearance, and health, making body-related experiences more prominent in their psychological lives. In this context, body appreciation is particularly significant in later life and may be associated with life satisfaction (Wodarz and Rogowska, 2024). For older adults, body-related attitudes are often linked to broader psychological experiences. As they face physical changes and aging, their self-attitudes and perceptions of aging are closely related to life evaluations (El-Sayed et al., 2024; Li et al., 2021; Zhao et al., 2026). So, self-related experiences and aging perceptions are key to understanding psychological differences among older adults.

The positive body image framework, rooted in positive psychology, emphasizes viewing one’s body with acceptance, respect, and gratitude. It considers positive body image as a positive psychological construct, independent of negative body image (Tylka and Wood-Barcalow, 2015). From this perspective, body appreciation is not only satisfaction with appearance or function but also involves integrating body experiences into self-evaluation. This process is linked to higher self-acceptance and life satisfaction (Matera et al., 2024; Swami et al., 2024). In later life, changes in physical function and health become more evident, making body-related experiences increasingly prominent in daily psychological life (Wilton-Harding and Windsor, 2022). Based on this view, the positive body image framework provides a clear conceptual starting point for understanding the psychological meaning of body appreciation among older adults and its associations with self-acceptance and life satisfaction.

The subjective aging framework suggests that aging is not just a physiological process but also how individuals perceive, evaluate, and experience their age-related changes (Diehl et al., 2014). Awareness of these changes and attitudes toward aging are crucial aspects of older adults’ self-experience. These factors are consistently associated with life satisfaction, well-being, and mental health, key indicators of subjective well-being (Diehl and Wahl, 2024; Velaithan et al., 2024). The extended subjective aging framework emphasizes that self-perceptions of aging (SPA) are not just reflections of aging experiences but are also connected to individuals’ self-evaluation systems (Sabatini et al., 2025). In this context, body appreciation, as part of self-evaluation, may be linked to SPA and life satisfaction (Nakajima and Kinjo, 2025).

Previous studies provide evidence for understanding the relationship between positive body attitudes and well-being outcomes. In general adult and young populations, positive body attitudes are associated with psychological well-being and life satisfaction. However, direct evidence in older adults is limited (Martone et al., 2025; Matera et al., 2024). In contrast, older adults experience more noticeable physical changes and aging-related subjective experiences. Thus, it remains to be seen whether the association between body appreciation and life satisfaction also holds, and through which psychological processes it occurs, particularly in samples more relevant to older adults’ daily experiences.

While direct evidence in older adults is limited, some studies suggest a stable association between body appreciation and life satisfaction in this group (Swami and Todd, 2022; Wodarz and Rogowska, 2024). For example, a study by Wodarz and Rogowska (2024), which covered stages from early adulthood to older age, showed that body appreciation and life satisfaction are positively linked across age groups. This association does not show clear weakening or reversal in later adulthood. In addition, a study by Swami and Todd (2022) also showed a significant positive association between body appreciation and life satisfaction. Their analysis also examined the link between place of residence (rural or urban) and life satisfaction. This provides additional evidence for the stability of this association across different living contexts. These findings suggest that even in stages with more noticeable physical changes, positive attitudes toward the body among older adults remain stably related to their overall evaluations of life. Overall, existing studies provide initial empirical support for understanding the relationship between body appreciation and life satisfaction among older adults. However, related evidence still needs further accumulation and refinement. Therefore, Hypothesis H1 is proposed: a significant association exists between body appreciation and life satisfaction.

Self-acceptance usually refers to individuals’ overall accepting attitude toward themselves. It includes recognition of personal strengths and tolerance of personal limitations (Li et al., 2025). In later life, self-acceptance is more reflected in a calm attitude toward changes in physical function, role transitions, and adjustments in daily rhythm. It also involves maintaining relatively stable self-worth and self-reconciliation under these changes (Achwandi, 2025). Studies based on different groups of older adults have found a significant association between self-acceptance and life satisfaction (Alfonso-Benlliure et al., 2021; Li et al., 2021). For example, Li et al. (2021) used older adults in Chinese nursing homes as their sample. They reported a strong link between self-acceptance and subjective well-being. This indicator of subjective well-being included overall evaluations of life. These results suggest that self-acceptance is closely related to overall life evaluations among older adults. Similarly, Alfonso-Benlliure et al. (2021) tested a structural model of psychological well-being and life satisfaction in a sample aged 65 to 84. They included self-acceptance as an important dimension of psychological well-being. Their results showed that self-acceptance was notably associated with life satisfaction. This further supports the close association between self-acceptance and life satisfaction in older age. Overall, existing studies have repeatedly observed significant associations between the two variables in different samples of older adults. They also suggest at the structural level that self-acceptance may be an important self-evaluation characteristic for understanding differences in life satisfaction among older adults. Therefore, Hypothesis H2 is proposed: a significant association exists between self-acceptance and life satisfaction.

Body appreciation is a positive body attitude. Previous studies show that it is associated with more positive self-evaluations. Older adults with higher levels of body appreciation usually report higher self-acceptance (Matera et al., 2024; Swami et al., 2024). For example, Matera et al. (2024) measured body appreciation and self-acceptance in a sample aged 18 to 81. Using path analysis, they found a significant association between the two variables. This shows that body appreciation and self-acceptance present stable positive links within the same statistical model. In addition, Swami et al. (2024) found that body appreciation was significantly related to “positive self-beliefs.” The measurement of this construct clearly included self-evaluation components such as self-acceptance. This provides further evidence, at the level of overall self-beliefs, for the association between body appreciation and self-acceptance. These studies together show that whether self-acceptance is measured directly or reflected as part of broader positive self-beliefs, body appreciation shows consistent associations with more positive self-evaluation tendencies. This suggests that body attitudes and overall self-attitudes have relatively stable empirical links. Therefore, Hypothesis H3 is proposed: a significant association exists between body appreciation and self-acceptance.

At present, few studies among older adults have directly used self-acceptance as a mediating variable to systematically examine the relationship between body appreciation and life satisfaction. Nevertheless, current studies have provided relatively consistent empirical support at the level of pairwise associations. Therefore, Hypothesis H4 is proposed: self-acceptance mediates the relationship between body appreciation and life satisfaction.

SPA usually refer to individuals’ subjective evaluations and experiences of their own aging process and related changes. They reflect individuals’ attitudes toward aging (Diehl and Wahl, 2024). Previous studies consistently show associations between SPA and life satisfaction among older adults. More positive SPA are often accompanied by higher life satisfaction (Che et al., 2024; Yuan et al., 2024). For example, based on longitudinal data from older adults in China, Yuan et al. (2024) found that more positive SPA showed significant statistical associations with later levels of life satisfaction. This association could also be observed in cross-lagged analyses. These findings suggest consistency between aging-related self-evaluations and life evaluations across time. Similarly, Che et al. (2024) found in a community sample of middle-aged and older adults in Macao that multidimensional SPA were significantly positively related to life satisfaction. These relationships showed clear statistical structures within a multiple mediation framework. This indicates that older adults’ subjective evaluations of aging experiences often change in parallel with their overall evaluations of life quality. Overall, according to these investigations, SPA are not temporary attitudes. Instead, they represent psychological patterns that remain relatively stable and parallel with subjective well-being evaluations such as life satisfaction. Therefore, Hypothesis H5 is proposed: a significant association exists between SPA and life satisfaction.

From the perspective of aging development, physical changes are an important practical basis for the formation of SPA. So, body-related attitudes may be associated with older adults’ SPA. Although empirical studies that directly focus on body appreciation and SPA among older adults remain relatively limited, existing evidence has suggested consistent statistical links between the two variables in older samples. For example, El-Sayed et al. (2024) found significant associations between body appreciation and SPA in a community-dwelling sample of women aged 60 and above. They also found that these two variables showed systematic statistical links with psychological variables such as self-efficacy and cognitive flexibility. These findings imply that within this sample, older adults with more positive attitudes toward their bodies often also express more optimistic views about getting older. This provides support for empirical associations between body experiences and SPA. Therefore, Hypothesis H6 is proposed: a significant association exists between body appreciation and SPA.

Similarly, based on the existing literature, few studies have directly used SPA as a mediating variable to systematically examine the relationship between body appreciation and life satisfaction. Nevertheless, previous research has provided some empirical support at the level of pairwise associations. Therefore, Hypothesis H7 is proposed: SPA mediate the relationship between body appreciation and life satisfaction.

**Figure 1:**
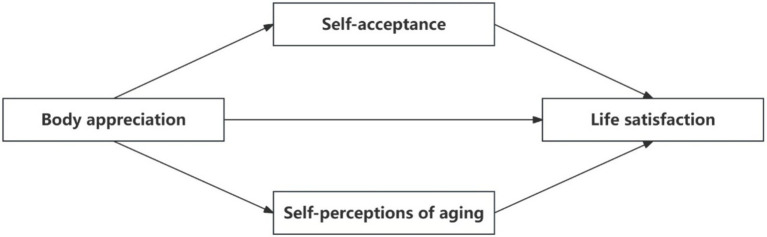
Research model.

Based on previous studies, this study integrates the positive body image framework and the subjective aging framework. It focuses on older adults and examines the association mechanisms between body appreciation and life satisfaction. It also includes self-acceptance and SPA in the model as possible mediating variables between the two. Based on this, the study constructs a conceptual model (see Figure 1).

## 2 Methods

### 2.1 Sample and data collection

Data for this study were collected from November 4 to December 27, 2025. The participants were older adults aged 60–80 years from Nantong City, Jiangsu province. According to the Seventh National Census, individuals aged 60–80 account for 24.67% of the permanent resident population in this region, providing an adequate sampling base for this study. The research team randomly selected older adults across three districts, including Chongchuan district, Haimen district, and Tongzhou district. These communities served as supporting units for the dissemination of the online questionnaire. This study adopted an online questionnaire survey. The research team distributed the survey link or QR code through community committees, community health service centers, senior universities, and senior interest groups. The survey was also shared on online platforms commonly used by older adults, such as WeChat groups and public accounts. Participants were encouraged to forward the survey link to other eligible individuals to broaden the range of participants. This approach helped include older adults from different community backgrounds as well as those active on online platforms, increasing the geographical and social diversity of the sample. The inclusion criteria were that participants had to be able to understand the questionnaire content and voluntarily agree to participate. To ensure smooth completion of the survey, the research team provided online or phone guidance for technical operation when needed, especially for those who had difficulties using electronic devices. This assistance was limited to operational instructions and did not involve questionnaire content or influence participants’ responses. Before completing the questionnaire, all participants read the study information online. They entered the formal survey only after confirming informed consent by selecting the required option. According to the sample size estimation principle proposed by Kline (2018), which is based on 10 times the number of measurement items and includes about 20% additional samples to address invalid questionnaires, this study planned to collect no fewer than 588 valid samples. Finally, this study collected a total of 627 questionnaires. According to the scale data quality control principles proposed by Boateng et al. (2018) the research team conducted systematic screening of all questionnaires during the data processing stage. Specifically, the study removed questionnaires with missing key items and those showing abnormal response patterns. These patterns included repeatedly selecting the same option across many items, highly repetitive response styles, or clear logical inconsistencies between items. After these quality control procedures, the study obtained 605 valid questionnaires for subsequent statistical analyses. The sample size met the research requirements.

### 2.2 Measures

In this study, all scales used a five-point Likert scale. The original structure and core content of each scale remained unchanged. Response options ranged from 1 (strongly disagree) to 5 (strongly agree). Higher scores indicated higher levels of the corresponding variables.

To assess participants’ levels of body appreciation, this study used the Body Appreciation Scale translated and validated in the Chinese cultural context Swami et al. (2016). This scale contains 10 items, such as “I appreciate the unique and different shapes of my body.” The Cronbach’s *α* was 0.925.

To assess participants’ levels of self-acceptance, this study used the Self-Acceptance Questionnaire developed by Xie (2016) for older adults in China. This questionnaire contains 13 items, such as “I admit and accept the shadow in my heart” (Li et al., 2021). The Cronbach’s α was 0.932.

To assess participants’ levels of positive attitudes toward aging, this study used the brief version of the Attitudes to Ageing Questionnaire revised for the Chinese cultural context by Gao et al. (2024). This questionnaire contains 12 items, such as “Physical health problems do not prevent me from doing what I want to do” (Liu et al., 2025). The Cronbach’s α was 0.924.

To assess participants’ levels of life satisfaction, this study used the Satisfaction With Life Scale developed by Diener et al. (1985). This questionnaire contains five items, such as “So far, I have gotten the important things I want in life.” It shows good reliability and validity in China (Hou et al., 2022). The Cronbach’s α was 0.863.

### 2.3 Statistical analysis

All statistical analyses were conducted using SPSS 26.0. First, the study calculated descriptive statistics and conducted correlation analyses. It also examined the possibility of common method bias and assessed levels of multicollinearity. These steps were used to determine whether the data met the basic requirements for multivariate analysis. Then, the study used multiple regression to examine the statistical associations among body appreciation, self-acceptance, SPA, and life satisfaction. This study employed the PROCESS macro (Model 4) for analysis in order to investigate the simultaneous mediating effects. Indirect effects and their confidence intervals were estimated using 5,000 bootstrap samples. This approach was used to improve the robustness and credibility of the results.

## 3 Results

### 3.1 Measurement model

The results of the single-factor test for common method bias showed that the variance explained by the first common factor extracted in the unrotated factor analysis was 35.699%, which did not exceed the empirical threshold of 40%. The results of the simultaneous latent variable method showed that the chi-square difference between the control model and the baseline model after adding the common method latent variable was not significant (*p* > 0.05), indicating that the common method bias was unlikely to have a substantial impact on the research results in this study (Williams et al., 2010).

Linear regression was conducted to test for multicollinearity. VIF values were low, with a maximum of 1.689. This value was below 3 (Table 1), and multicollinearity did not appear to be a key issue (Gómez et al., 2020).

**Table 1:** Collinearity test.

Independent variable	Tolerance	VIF
BA	0.592	1.689
SA	0.752	1.331
SPA	0.738	1.355

BA, Body appreciation; SA, Self-acceptance; SPA, Self-perceptions of aging.

Table 2 displays the findings of this study, which evaluated the scale’s dependability using measurement model analysis. Except for SPA 4, the loading of all items exceeded 0.708, and the composite reliability (CR) of all constructs was higher than 0.708. The average variance extracted (AVE) of each dimension is higher than 0.5, indicating that the model has good reliability and validity (Joseph F. Hair et al., 2021). In addition, according to the retention criteria of Joseph F. Hair et al. (2021), when the remaining indicators have reached the standard and the item is theoretically necessary, the load of 0.60–0.70 with a value of 0.705 is still acceptable, so SPA 4 is retained.

**Table 2:** Reliability and validity.

Constructs	Items	Loadings	CR	Cronbach’s α	AVE
BA	BA 1	0.802	0.936	0.925	0.596
	BA 2	0.759			
	BA 3	0.790			
	BA 4	0.806			
	BA 5	0.775			
	BA 6	0.759			
	BA 7	0.764			
	BA 8	0.718			
	BA 9	0.768			
	BA 10	0.776			
SA	SA 1	0.744	0.941	0.932	0.553
	SA 2	0.764			
	SA 3	0.730			
	SA 4	0.741			
	SA 5	0.748			
	SA 6	0.751			
	SA 7	0.714			
	SA 8	0.781			
	SA 9	0.747			
	SA 10	0.736			
	SA 11	0.748			
	SA 12	0.723			
	SA 13	0.733			
SPA	SPA 1	0.750	0.936	0.924	0.549
	SPA 2	0.736			
	SPA 3	0.725			
	SPA 4	0.705			
	SPA 5	0.745			
	SPA 6	0.709			
	SPA 7	0.732			
	SPA 8	0.755			
	SPA 9	0.715			
	SPA 10	0.728			
	SPA 11	0.766			
	SPA 12	0.820			
LS	LS 1	0.807	0.902	0.863	0.647
	LS 2	0.801			
	LS 3	0.806			
	LS 4	0.809			
	LS 5	0.798			

BA, Body appreciation; SA, Self-acceptance; SPA, Self-perceptions of aging; LS, Life satisfaction.

### 3.2 Comparison of life satisfaction scores across different demographic characteristics

The descriptive statistics of the sample are shown in Table 3. Life satisfaction shows relatively clear stratified differences across different demographic groups. The results of descriptive analysis showed that there were significant differences in life satisfaction among different demographic groups, mainly in terms of age, education level, marital status, place of residence, satisfaction with economic status and health status (*p* < 0.001). In general, the older adults with better socio-economic conditions and health status showed a more favorable distribution of life satisfaction.

**Table 3:** Demographic characteristics of the sample.

Demographic characteristic	Category	Quantity	Percentage	Life satisfaction	
				*T/F*	*p*
Gender	Male	305	50.4%	−1.025	0.306
	Female	300	49.6%		
Age	60–70	332	54.9%	13.760	< 0.001
	71–80	273	45.1%		
Education level	Primary school and below	292	48.3%	187.006	< 0.001
	Junior high school	156	25.8%		
	Senior high school	89	14.7%		
	College and above marital status	68	11.2%		
Marital status	Married	435	71.9%	81.502	< 0.001
	Widowed	127	21.0%		
	Divorced	43	7.1%		
Hometown	Urban	319	52.7%	23.034	< 0.001
	Rural	286	47.3%		
Satisfaction with financial situation	Very satisfied	95	15.7%	236.703	< 0.001
	Satisfied	143	23.6%		
	Neutral	131	21.7%		
	Dissatisfied	132	21.8%		
	Very dissatisfied	104	17.2%		
Participation in pension insurance	Yes	444	73.4%	4.354	< 0.001
	No	161	26.6%		
Participation in medical insurance	Yes	537	88.8%	−0.828	0.408
	No	68	11.2%		
Health status	Very good	105	17.4%	86.842	< 0.001
	Good	134	22.1%		
	Average	150	24.8%		
	Poor	102	16.9%		
	Very poor	114	18.8%		

### 3.3 Correlation between variables

The results of correlation analysis showed that the research variables presented a consistent and significant correlation pattern (Table 4). Body appreciation was significantly and positively correlated with self-acceptance, SPA and life satisfaction. Self-acceptance was also significantly and positively correlated with SPA, and both were significantly and positively correlated with life satisfaction. Overall, the correlation direction between the variables was consistent with the theoretical expectation, and the correlation degree was at a medium level, without excessive correlation, which provided a good statistical basis for the subsequent mediation model analysis.

**Table 4:** Correlation analysis.

Variables	M ± SD	BA	SA	SPA	LS
BA	3.277 ± 0.922	1			
SA	3.247 ± 0.903	0.498***	1		
SPA	3.199 ± 0.868	0.512***	0.252***	1	
LS	3.223 ± 0.964	0.582***	0.602***	0.535***	1

***p < 0.001; BA, Body appreciation; SA, Self-acceptance; SPA, Self-perceptions of aging; LS, Life satisfaction.

### 3.4 The mediating role of self-acceptance and SPA

After controlling for demographic variables such as age, education level, marital status, place of residence, satisfaction with economic status, pension insurance, and health status, the regression results are shown in Table 5. Body appreciation is significantly positively correlated with self-acceptance and SPA; at the same time, body appreciation is still significantly positively correlated with life satisfaction. After further including self-acceptance and SPA into the regression equation of life satisfaction at the same time, self-acceptance is significantly positively correlated with life satisfaction, while the relationship between SPA and life satisfaction does not reach a significant level, suggesting that after strictly controlling demographic factors, the independent contribution of SPA to life satisfaction is relatively limited.

**Table 5:** Regression analysis of the mediation effect.

Outcome variable	Predictor variable	*β*	*T*	95% confidence interval		*R* ^2^	*F*
				Lower bound	Upper bound		
SA	BA	0.268	6.594***	0.189	0.348	0.366	42.997
	Age	−0.004	−0.058	−0.137	0.129		
	Education level	0.094	2.421*	0.018	0.171		
	Marital status	−0.034	−0.612	−0.143	0.075		
	Hometown	−0.225	−3.035**	−0.371	−0.079		
	Satisfaction with financial situation	−0.164	−5.242***	−0.225	−0.102		
	Participation in pension insurance	0.118	1.710	−0.018	0.253		
	Health status	−0.010	−0.380	−0.062	0.042		
SPA	BA	0.231	6.735***	0.164	0.299	0.511	77.914
	Age	−0.334	−5.967***	−0.452	−0.228		
	Education level	−0.021	−0.628	−0.085	0.044		
	Marital status	−0.403	−8.628***	−0.495	−0.311		
	Hometown	−0.065	−1.045	−0.188	0.058		
	Satisfaction with financial situation	−0.004	−0.145	−0.056	0.048		
	Participation in pension insurance	0.042	0.721	−0.072	0.156		
	Health status	−0.166	−7.405***	−0.211	−0.122		
LS	BA	0.061	2.434*	0.012	0.110	0.823	275.249
	SA	0.147	6.244***	0.101	0.193		
	SPA	0.031	1.122	−0.024	0.086		
	Age	−0.152	−3.857***	−0.229	−0.075		
	Education level	0.192	8.689***	0.149	0.236		
	Marital status	−0.111	−3.311**	−0.176	−0.045		
	Hometown	−0.439	−10.373***	−0.523	−0.356		
	Satisfaction with financial situation	−0.225	−12.442***	−0.260	−0.189		
	Participation in pension insurance	0.008	0.210	−0.069	0.085		
	Health status	−0.096	−6.059***	−0.127	−0.065		

*p < 0.05, **p < 0.01, ***p < 0.001; BA, Body appreciation; SA, Self-acceptance; SPA, Self-perceptions of aging; LS, Life satisfaction.

Based on this, the results of the mediation effect test in Table 6 show that the total effect of body appreciation on life satisfaction reached a significant level. After the inclusion of the mediating variables, the direct effect was still significant but weakened compared to the total effect, while the total indirect effect was significant, accounting for 43.5% of the total effect. In summary, the main contribution of the mediating effect is more concentrated on the path of self-acceptance, while the path of SPA did not show a stable independent predictive effect after controlling for demographic variables. It should be pointed out that after the inclusion of the above demographic variables as control variables, the effect of each path was weakened, but it remained significant. This result shows that some of the effects are related to the differences in life resources reflected by demographic factors, and after strictly controlling these factors, the association between the research variables is still statistically significant, showing the robustness of the results.

**Table 6:** The test results of the mediating effect.

Path	*β*	*SE*	95% confidence interval		Percentage
			Lower bound	Upper bound	
Total effect	0.108	0.024	0.061	0.154	100%
Direct effect	0.061	0.025	0.012	0.110	56.5%
Indirect effect					
SA	0.040	0.009	0.023	0.059	37.0%
SPA	0.007	0.007	−0.005	0.020	6.5%

SA, Self-acceptance; SPA, Self-perceptions of aging.

## 4 Discussion

From the demographic results of this study, differences in life satisfaction among older adults were more closely related to whether daily life runs smoothly rather than to gender. The gender difference was not significant, suggesting that in later life, life evaluations may be more centered on common practical factors such as health status, support systems, and institutional security. In contrast, the 60–70 age group reported higher life satisfaction than the 71–80 group, and a gradient pattern was observed across levels of health status. This pattern reflects a gradual narrowing of functional capacity and life space with increasing age. When mobility declines or chronic conditions accumulate, independence and daily sense of control may decrease, and life satisfaction is more likely to be lower (Zhang et al., 2022). The gradient differences in education level, urban–rural residence, and satisfaction with economic status further point to resource accessibility. Older adults with higher education, those living in urban areas, and those more satisfied with their economic condition generally have greater access to medical services, information, and community support, and are more likely to maintain a stable daily routine and subjective sense of satisfaction (Xie et al., 2024; Yang and Zhang, 2024). Regarding marital status, married older adults reported higher life satisfaction, reflecting the role of partners in companionship and practical support, whereas widowhood or divorce may be accompanied by a weakened support network. The significant difference in pension insurance also indicates the importance of institutional security for life evaluation in later life (Xia et al., 2024). In contrast, the difference in medical insurance was not significant, possibly due to its relatively high coverage rate. Overall, the results of this study indicate that life satisfaction among older adults is more closely associated with functional health status, resource accessibility, and stable support networks rather than with single demographic labels.

The results of this study show a positive association between body appreciation and life satisfaction among older adults. This finding is consistent with existing studies (Swami and Todd, 2022; Wodarz and Rogowska, 2024). This association is not only reflected in more optimistic attitudes. It is also linked with lower self-devaluation and higher self-recognition. These patterns are often accompanied by more positive evaluations of life (Wodarz and Rogowska, 2024). When older adults are more appreciative of their bodies, they are also more willing to accept the changes that come with age, such as wrinkles, changes in body shape, decreased physical strength, or chronic discomfort. As a result, they are less likely to fall into self-denial when looking in the mirror and dressing up. They are also less likely to avoid going out to socialize because they “feel indecent.” On the contrary, this sense of body recognition is more likely to allow them to maintain a more leisurely pace of life (Syue et al., 2022; Wu et al., 2024). For example, they are more willing to go out for a walk, bask in the sun, participate in community activities, or take more initiative to take care of their sleep and diet. Over time, these seemingly insignificant things will accumulate into a more stable sense of comfort and control, making them more satisfied with their overall evaluation of life.

After controlling for age, education level, marital status, urban or rural residence, satisfaction with economic status, pension insurance, and health status, self-acceptance still showed a clear mediating pattern between body appreciation and life satisfaction. The indirect effect accounted for 37.0% of the total effect, consistent with previous studies (Li et al., 2021; Matera et al., 2024). In daily life, life satisfaction among older adults does not depend on the complete absence of physical discomfort, but rather on whether individuals can live in harmony with these changes. Many older adults repeatedly experience pain, fatigue, slower walking, or lighter sleep, and even changes in appearance may be casually mentioned by others. Those with higher body appreciation tend to interpret these changes in a gentler way and evaluate themselves less harshly, allowing self-acceptance to remain at a higher level (Swami et al., 2024). Here, self-acceptance functions as a stable psychological stance. It allows older adults to view physical changes as part of the life process rather than as personal failure. This stable self-attitude makes them less sensitive in family interactions and less likely to withdraw from social activities due to self-denial, helping life satisfaction remain relatively stable (Li et al., 2021). The indirect effect of 37.0% indicates that this pathway retains practical weight even after demographic factors are included. For many older adults, external conditions such as health and economic status are difficult to change in the short term. Therefore, maintaining dignity and a sense of self-worth amid irreversible physical changes is closely related to the stability of life experiences (Yan et al., 2024). At the same time, the direct effect accounted for 56.5%, suggesting that self-acceptance does not explain all associations between body appreciation and life satisfaction. The remaining portion may stem from more direct experiences, such as confidence in physical functioning and a sense of control over daily rhythm. This indicates that life satisfaction is supported by multiple overlapping pathways, with self-acceptance being an important one.

After controlling for age, education level, marital status, urban or rural residence, satisfaction with economic status, pension insurance, and health status, SPA did not show a mediating pattern between body appreciation and life satisfaction. This finding is not fully consistent with previous studies (El-Sayed et al., 2024; Yuan et al., 2024). Statistically, SPA showed significant positive associations with both body appreciation and life satisfaction. However, when body appreciation, self-acceptance, and demographic factors were included simultaneously, SPA did not provide significant independent explanatory value. In other words, more positive SPA is indeed accompanied by higher life satisfaction, but its additional contribution becomes limited when multiple variables are considered together. This does not imply that SPA is unimportant. Rather, it may indicate that the portion of life satisfaction associated with SPA overlaps with factors more closely tied to daily life experiences (Sabatini et al., 2025). From a life-context perspective, SPA reflects a general form of self-understanding, including acceptance of aging and a more positive view of age-related changes. It may help older adults maintain inner stability, but it may not directly correspond to satisfaction with specific aspects of daily life. Life satisfaction is often gradually accumulated through concrete experiences, such as sleep quality, pain levels, self-care ability, travel convenience, and social support (Sabatini et al., 2025; Sulandari et al., 2024). In addition, the role of SPA may be context-dependent. Among older adults with better health and greater independence, positive SPA may be more visible in stable life experiences. In contrast, among those with poorer health or higher care needs, this association may be less apparent and more easily overshadowed by practical difficulties (Che et al., 2024). So, after controlling for key practical factors such as health and economic satisfaction, the independent explanatory space of SPA becomes smaller, and it may function more as a supportive psychological resource.

In light of the findings of this study, the observed pattern may also be related to the specific socio-cultural context of China. Compared with Western cultures that emphasize individual independence, Chinese culture places greater importance on family connections and intergenerational support, and many older adults’ sense of self-worth remains embedded in family roles (Yuan et al., 2025). Therefore, body appreciation and self-acceptance are linked not only to internal evaluation but also to role stability within family and community contexts. When changes in physical function affect family participation, life evaluation is more likely to fluctuate (Zhou et al., 2025). This context may help explain why self-acceptance showed a stable mediating role, whereas SPA did not provide independent explanatory value. In a setting that emphasizes functional capacity and family roles, self-evaluative dimensions closely tied to daily experience may show stronger associations. In addition, under rapid population aging and urban–rural disparities, institutional security and resource access become more prominent, making functional health, support networks, and security structures more central in life evaluation.

## 5 Implications and limitations

### 5.1 Implications

The theoretical value of this study is in examining life satisfaction among older adults in a real-world context where body and aging experiences coexist. It also integrates the positive body image framework and the subjective aging framework within the same explanatory system. The findings indicate that body appreciation continues to be significantly linked to life satisfaction after accounting for demographic variables such as age, education, marital status, urban or rural residence, satisfaction with economic status, pension insurance, and health status. This finding extends the theoretical scope of positive body image research and suggests that its explanatory relevance is not limited to younger groups but also applies in later stages of the life course. At the same time, this study examines self-acceptance and SPA in parallel within the same model and distinguishes their theoretical orientations. Self-acceptance focuses on individuals’ overall acceptance of the self, while SPA focus on individuals’ subjective understanding of aging experiences. The results show that self-acceptance shows a mediating role between body appreciation and life satisfaction, whereas SPA do not show a mediating role in the multivariable model. This difference provides more specific clues for theoretical integration. It suggests that when explaining life satisfaction among older adults, self-attitude-related experiences and aging-related experiences may correspond to different positions in the association pathways. Therefore, these experiences need to be clearly distinguished in model construction and theoretical reasoning.

In combination with the results of this study in the older adults, a more feasible direction in practice is to implement the improvement of body appreciation and the promotion of self-acceptance in daily services and community scenarios, and help the older adults regard physical changes as part of life thru low-cost and sustainable ways, rather than equating it with the decline of self-worth. Communities and older adults care institutions can add simple positive physical experience training on the basis of health education, such as carrying out a light group activity once a week, focusing on the setting of small goals of physical function (such as walking distance, sleep regularity, pain self-management, etc.), physical gratitude practice (recording what the body has completed today), and alternative expression practice of negative self-evaluation, and gradually guide the older adults to shift their attention from what they used to be able to do to what they can still do. Self-acceptance can be used as the starting point for psychological support in front-line services. Short communication skills can be used in conjunction with chronic disease follow-up, rehabilitation training or home visits to help the older adults distinguish between physical changes and self-worth evaluation, reduce the emotional consumption caused by self-blame and avoidance, and thus make it easier to maintain social and daily activities. Given that life satisfaction shows clear differences across health status, satisfaction with economic status, urban or rural residence, and marital status, practice should prioritize more specific support for older adults who are older, in poorer health, less satisfied with their economic status, living in rural areas, or widowed or living alone. Examples include home-based rehabilitation guidance, pain and sleep management groups, neighbor support pairing, and convenient transport and appointment accompaniment services. These supports can help reduce daily barriers and support participation. For families and caregivers, the key is to avoid unintentional negative reminders, reduce expressions such as teasing about body shape or repeatedly emphasizing aging incompetence, and instead affirm functional progress and efforts, and encourage participation in accessible activities. Finally, SPA did not show a clear independent mediating effect in this study. This suggests that practice should not focus only on attitude promotion. It also suggests that resources should go to parts that are more directly tied to daily experiences. Examples include access to physical activity, practical steps for chronic disease management, ease of community participation, and sustainable strategies for body appreciation and self-acceptance. In this way, these efforts may be more likely to align with changes in life satisfaction that older adults can feel.

### 5.2 Limitations and future research directions

There are still some limitations in this study, which need to be further improved in the follow-up study. First, this study adopts a cross-sectional design, and the relationship between variables can only be tested for correlation and mediation structure at the same time point, which is difficult to reflect the dynamic process of change over time, and it is impossible to make a stronger inference on the directionality. Future studies can use longitudinal designs or cross-lagged models. They can measure body appreciation, self-acceptance, SPA, and life satisfaction at multiple time points. This approach can examine whether these associations remain stable. It can also show the trajectories of psychological experiences in later life more clearly. Second, this study collected data based on self-report questionnaires. Although it can efficiently obtain a large sample, it may still be affected by social desirability and recall bias. Especially in the older adults, differences in understanding and answering habits may further bring measurement errors. Future research can combine multi-source data and multi-method measurement, such as introducing other people’s evaluation or caregiver evaluation, interviews and diary methods, objective health indicators or activity volume records, etc., to improve the robustness of the conclusions. Third, the sample in this study has specific contextual features. Life satisfaction and related psychological variables may be related to regional resources, urban or rural differences, social support structures, and levels of social security. Therefore, the generalizability of the findings should be interpreted with caution. Future studies can conduct replication studies in different regions, across urban and rural settings, and under different pension security conditions. They can also use stratified sampling or multi-group comparisons to examine the applicability and boundary conditions of the model among different groups of older adults.

## 6 Conclusion

This study focused on older adults and combined the positive body image framework with the subjective aging framework. It examined the relationships among body appreciation, self-acceptance, SPA, and life satisfaction. The results show that after controlling for multiple demographic factors, body appreciation is significantly positively associated with life satisfaction, and self-acceptance shows a mediating role between the two. SPA show clear associations with body appreciation and life satisfaction at the correlational level. However, they do not show a clear independent mediating effect in the multivariable model. Overall, this study suggests that positive body experiences and self-attitudes in later life are closely related to life satisfaction. These findings provide theoretical support for enhancing life satisfaction among older adults and have significant practical implications. Interventions promoting body appreciation and self-acceptance can enhance life satisfaction and improve overall well-being in older adults. They may have certain applicability, especially in community and health service settings.

## Data Availability

The data that support the findings of this study are available from the corresponding author upon request. The raw data are also accessible through Zenodo Shi, L. (2026). Raw Data_An Examination of the Psychological Mechanisms between Body Appreciation and Life Satisfaction Among Older [Data set]. Zenodo. https://doi.org/10.5281/zenodo.18409634.
